# Supplementary material for: A targeted drug-repurposing strategy identifies Tavaborole (Kerydin) as a potent fungistatic agent against Candida auris
Source: Front Microbiol. 2026 Jun 12;17:1830629. doi: 10.3389/fmicb.2026.1830629 (PMC13303722; doi:10.3389/fmicb.2026.1830629)

Supplementary material 1

The primary *in-vitro* spotting assay utilized for the initial assessment of the inhibitory effect of compounds identified by the *in-silico* screening strategy. Assays were carried out using RPMI-MOPS with 2% glucose and 2% agar plates with final drug concentration of 50µM, *Candida auris* isolates belonging clade I (AR389), clade II (CBS10913), clade III (AR383), clade IV (AR385), clade V (AR1097), *Candida albicans* (SC5314) and *Candida glabrata* (ATCC2001) were used. Spotting was performed by a robotic arm, following which plates were monitored over time for fungal growth. The following compounds were screened: Carboxin (mw: 235.30), Cladribine (mw: 285.69), Colchicene (mw: 385.41), Dasatinib (mw: 488.01), Enasidenib (mw: 473.38), Fostamatinib (mw: 580.46), Griseofulvin (mw: 352.77), Mupirocin (mw: 500.62), Pemetrexed (mw: 427.41), Pimecrolimus (mw: 810.45), Selinexor (mw: 443.31), Sulfinpyrazone (mw: 404.12), Tavaborole (mw: 151.93), Triclabendazole (mw: 359.65) and additionally AmB 2µg/mL, control RPMI-MOPS and control YPD plates.

1. Carboxin 24h 48h

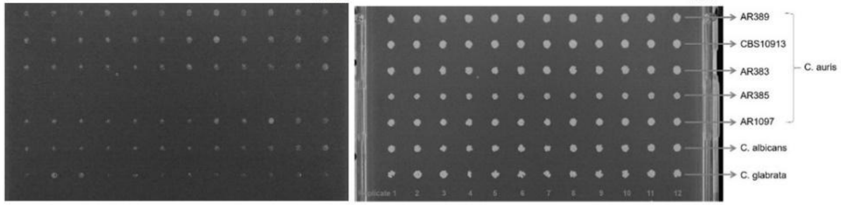

2. Cladribine 24h 48h

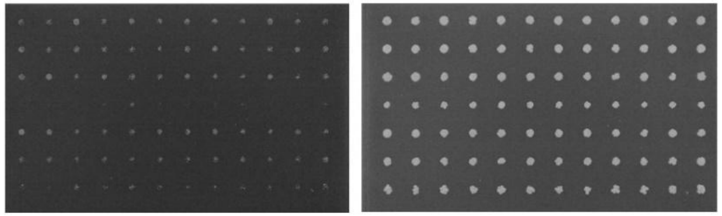

3. Colchicene 24h 48h

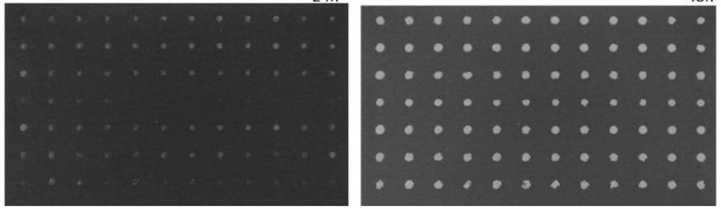

4. Dasatinib 24h 48h

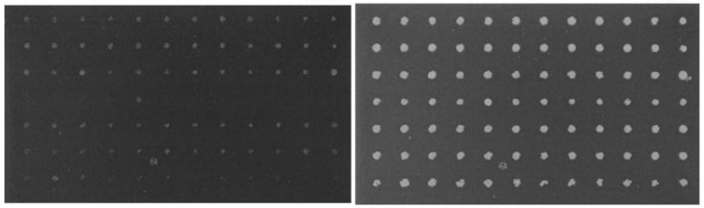

5. Enasidenib 24h 48h

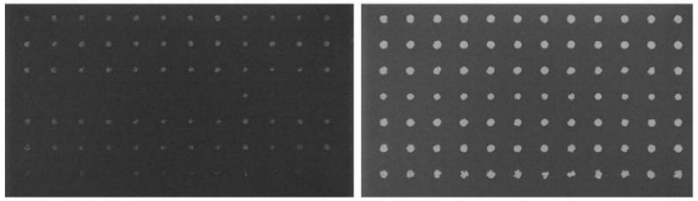

6. Fostamatinib 24h 48h

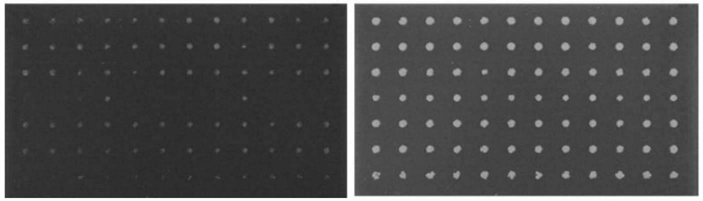

7. Griseofulvin

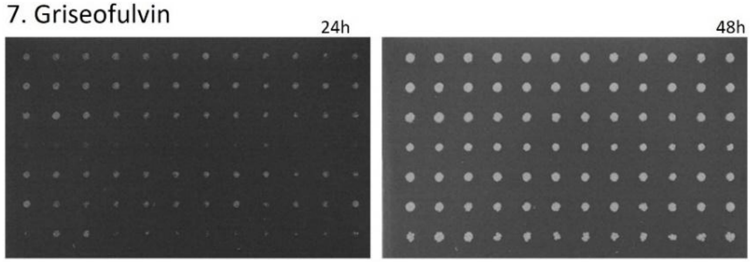

8. Mupirocin

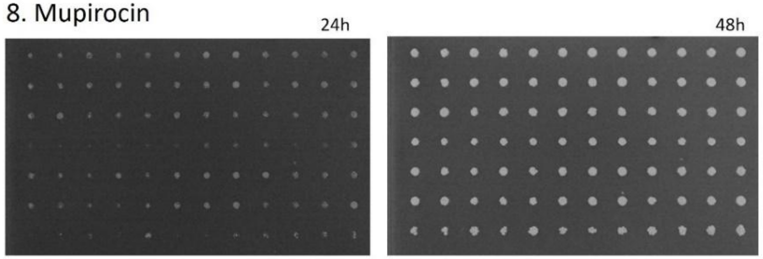

9. Pemetrexed

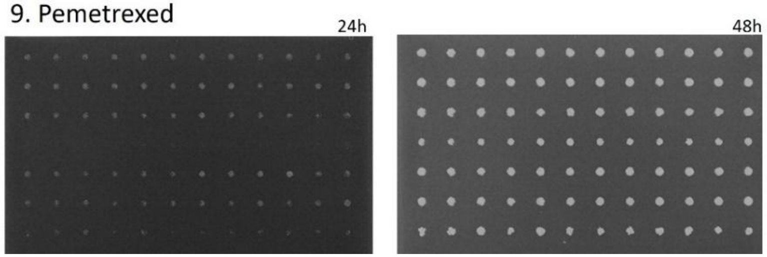

10. Pimecrolimus

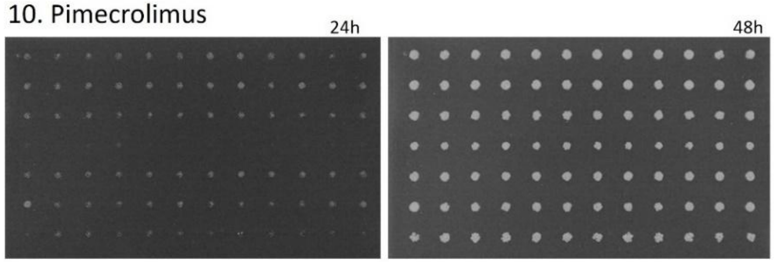

11. Selinexor

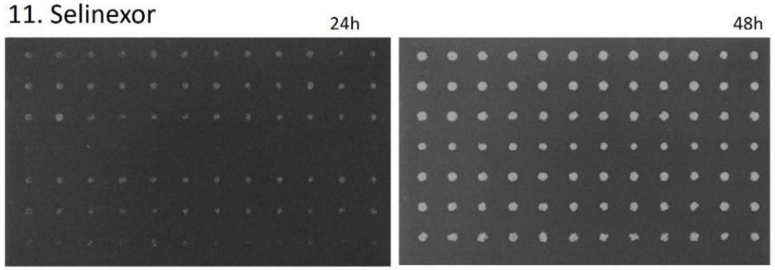

12. Sulfinpyrazone

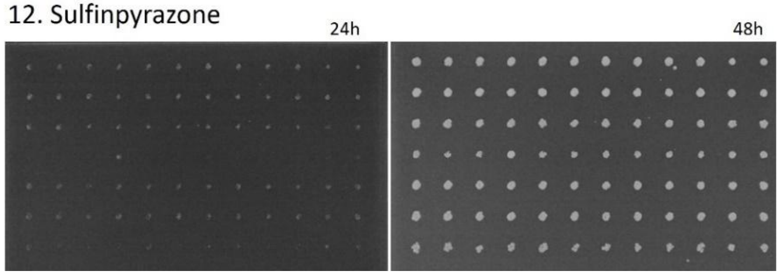

13. Tavaborole

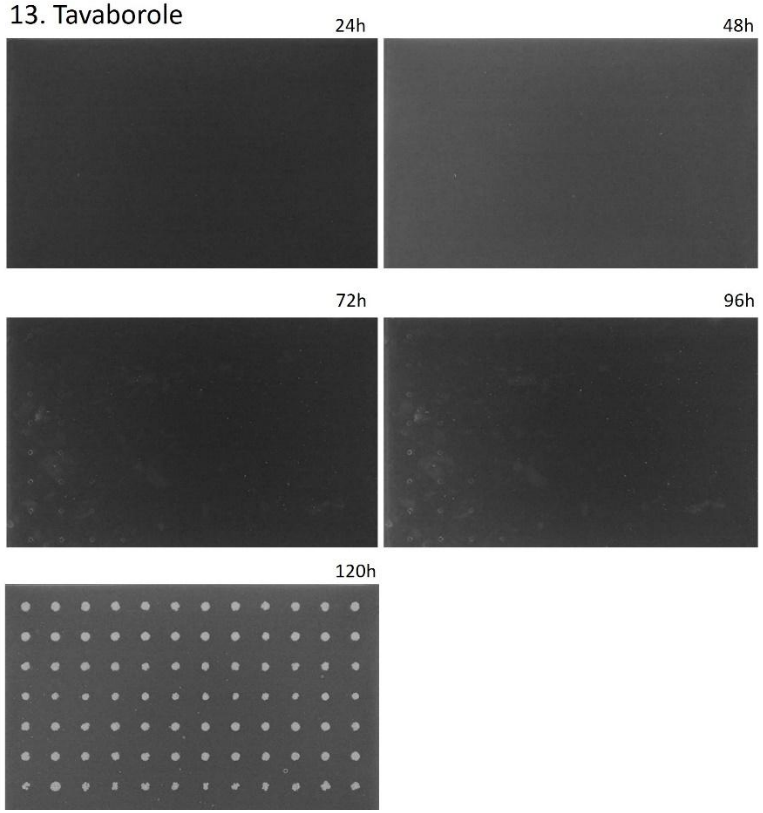

14. Triclabendazole

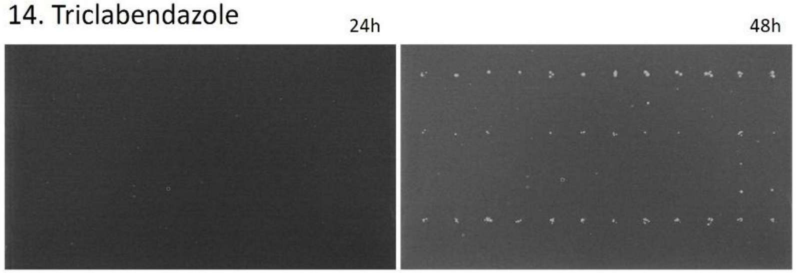

15. AmB

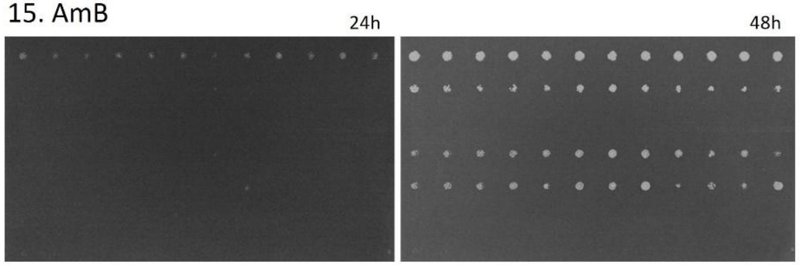

16. RPMI-MOPS control

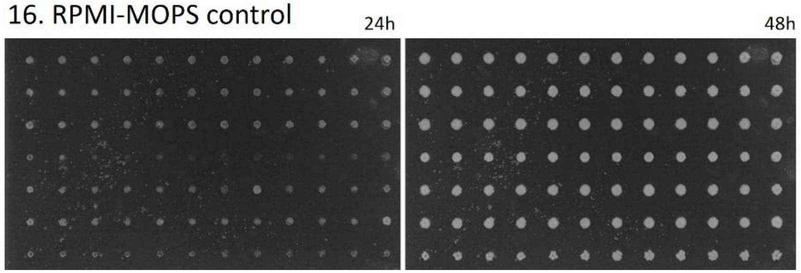

17. YPD control

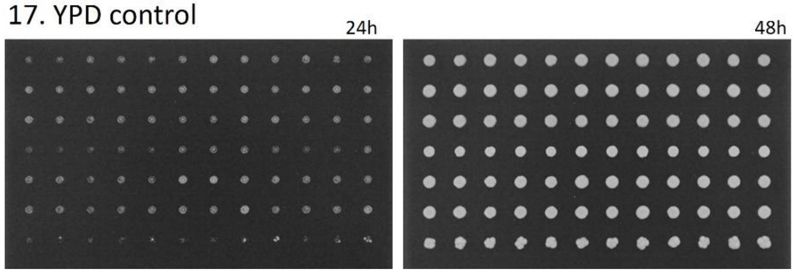

Supplement: Supplementary file 2 [file Data_Sheet_1.pdf]
